# 5-Chloro-*N*-(4,5-dihydro-1*H*-imidazol-2-yl)-2,1,3-benzothia­diazol-4-amine (tizanidine)

**DOI:** 10.1107/S1600536811008348

**Published:** 2011-03-12

**Authors:** Peter John, Islam Ullah Khan, Mehmet Akkurt, Muhammad Shahid Ramzan, Shahzad Sharif

**Affiliations:** a Materials Chemistry Laboratory, Department of Chemistry, GC University, Lahore 54000, Pakistan; b Department of Physics, Faculty of Sciences, Erciyes University, 38039 Kayseri, Turkey

## Abstract

There are two independent mol­ecules (*A* and *B*) with similar conformations in the asymmetric unit of the title compound, C_9_H_8_ClN_5_S. The benzothia­diazole ring systems of both mol­ecules are essentially planar [maximum deviation = 0.021 (2) Å in mol­ecule *A* and 0.022 (1) Å in mol­ecule *B*] and make dihedral angles of 68.78 (9) and 54.39 (8)°, respectively, with the mean planes of their 4,5-dihydro-1*H*-imidazole rings. An intra­molecular N—H⋯Cl hydrogen bond occurs in mol­ecule *B*. In the crystal, both mol­ecules form centrosymmetric dimers through π-stacking of their benzothia­diazole rings, with inter­planar distances of 3.3174 (7) and 3.2943 (6) Å. These dimers are further linked *via* pairs of N—H⋯N hydrogen bonds with the dihydro­imidazole rings as the hydrogen-bonding donors and one of the benzothia­diazole N atoms as the acceptors, generating *R*_2_^2^(16) ring motifs. The *A*_2_ and *B*_2_ dimers in turn form additional N—H⋯N hydrogen bonds with the secondary amine as the H-atom donor and the dihydro­imidazole N atom as the acceptor. These *R*_2_^2^(8)-type inter­actions connect the *A*_2_ and *B*_2_ dimers with each other, forming infinite chains along [1
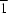
1].

## Related literature

For the medicinal importance of tizanidine, see: Koch *et al.* (1989 ▶); Shellenberger *et al.* (1999 ▶); Tse *et al.* (1987 ▶). For hydrogen-bond motifs, see: Bernstein *et al.* (1995 ▶).
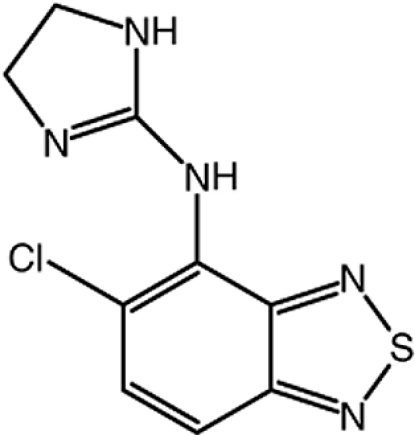


## Experimental

### 

#### Crystal data

C_9_H_8_ClN_5_S
*M* *_r_* = 253.72
Triclinic, 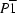

*a* = 7.6927 (3) Å
*b* = 10.8558 (4) Å
*c* = 12.9969 (5) Å
α = 95.790 (1)°
β = 101.126 (1)°
γ = 92.192 (1)°
*V* = 1057.69 (7) Å^3^
*Z* = 4
Mo *K*α radiation
μ = 0.54 mm^−1^
*T* = 296 K
0.29 × 0.18 × 0.08 mm

#### Data collection

Bruker APEXII CCD diffractometer
17897 measured reflections
5104 independent reflections
4449 reflections with *I* > 2σ(*I*)
*R*_int_ = 0.024

#### Refinement

*R*[*F* ^2^ > 2σ(*F* ^2^)] = 0.042
*wR*(*F* ^2^) = 0.120
*S* = 1.03
5104 reflections
302 parameters
5 restraints
H atoms treated by a mixture of independent and constrained refinement
Δρ_max_ = 0.65 e Å^−3^
Δρ_min_ = −0.43 e Å^−3^

### 

Data collection: *APEX2* (Bruker, 2007 ▶); cell refinement: *SAINT* (Bruker, 2007 ▶); data reduction: *SAINT*; program(s) used to solve structure: *SHELXS97* (Sheldrick, 2008 ▶); program(s) used to refine structure: *SHELXL97* (Sheldrick, 2008 ▶); molecular graphics: *ORTEP-3 for Windows* (Farrugia, 1997 ▶); software used to prepare material for publication: *WinGX* (Farrugia, 1999 ▶) and *PLATON* (Spek, 2009 ▶).

## Supplementary Material

Crystal structure: contains datablocks global, I. DOI: 10.1107/S1600536811008348/zl2351sup1.cif

Structure factors: contains datablocks I. DOI: 10.1107/S1600536811008348/zl2351Isup2.hkl

Additional supplementary materials:  crystallographic information; 3D view; checkCIF report

## Figures and Tables

**Table 1:** Hydrogen-bond geometry (Å, °)

*D*—H⋯*A*	*D*—H	H⋯*A*	*D*⋯*A*	*D*—H⋯*A*
N3*A*—H*N*3*A*⋯N5*B*	0.92 (2)	2.10 (2)	3.003 (2)	168 (3)
N4*A*—H*N*4*A*⋯N1*A*^i^	0.86 (3)	2.38 (3)	3.205 (3)	160 (2)
N3*B*—H*N*3*B*⋯N5*A*	0.88 (2)	1.98 (2)	2.864 (2)	177 (2)
N4*B*—H*N*4*B*⋯Cl1*B*	0.84 (2)	2.75 (2)	3.1927 (15)	114 (2)
N4*B*—H*N*4*B*⋯N1*B*^ii^	0.84 (2)	2.48 (2)	3.227 (2)	150 (2)

Symmetry codes: (i) 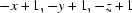; (ii) 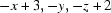.

**Fig. 1.**
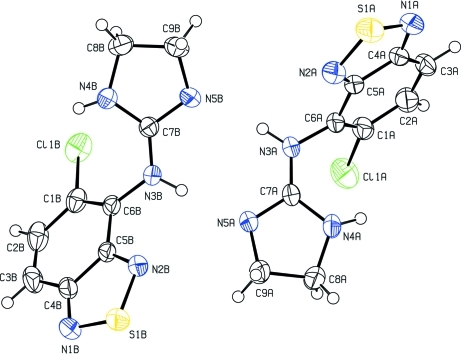
View of the two crystallographically independent molecules, A and B, with the atom numbering scheme. Displacement ellipsoids for non-H atoms are drawn at the 50% probability level.

**Fig. 2.**
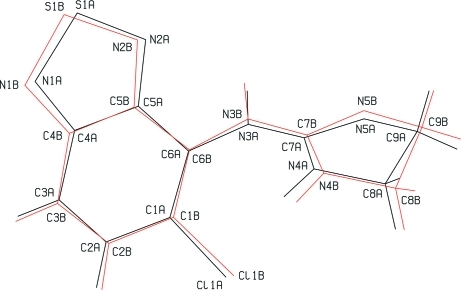
An overlay of the two molecules A (black line) and B (red line).

**Fig. 3.**
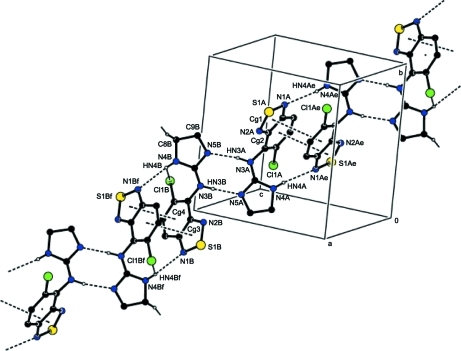
A view of the π-π stacking interactions and the hydrogen bonding of the title compound. The H atoms not involved in the hydrogen bonds forming the complete motif were omitted. (Symmetry codes: (e) 1-x, 1-y, 1-z; (f) 3-x, -y, 2-z).

## supplementary crystallographic information

### Comment

Tizanidine {or (*5-chloro-N-(4,5-dihydro-1H-imidazol-2-yl)-2,1,3-benzothiadiazol-4-amine)*} is an adrenergic agonist and proved to be an active myotonolytic skeletal muscle relaxant with a chemical structure different from other muscle relaxants (Koch *et al.*, 1989; Tse *et al.*, 1987). It also reduces increased muscle tone associated with spasticity in patients with multiple sclerosis or spinal cord injury (Shellenberger *et al.*, 1999). Herein, we report the crystal structure of Tizanidine.

The title compound crystallized with two unique molecules A and B in the asymmetric unit (Fig. 1). The benzothiadiazole ring systems (S1A/N1A/N2A/C1A–C6A and S1B/N1B/N2B/C1B) of both molecules A and B are essentially planar [max. deviations = 0.021 (2) Å for C5A in molecule A and 0.022 (1) Å for C6B in molecule B] and they form dihedral angles of 68.78 (9) and 54.39 (8)°, respectively, with the mean planes of their 4,5-dihydro-1*H*-imidazole rings (N4A/N5A/C7A–C9A and N4B/N5B/C7B–C9B). The conformations of molecules *A* and *B* are similar (Fig. 2).

Molecular conformations in the crystal structure are stabilized by intramolecular N—H···N and N—H···Cl interactions (Table 1). Both molecules A and B are forming centrosymmetric dimers through π-stacking of their benzothiadiazole rings with interplanar distances of 3.3174 (7) and 3.2943 (6) Å [*Cg*1···*Cg*2*^ii^* = 3.6026 (10) Å and *Cg*3···*Cg*4*^iv^* = 3.5096 (9) Å; symmetry codes: (*i*) 1-*x*, 1-*y*, 1-*z*; (*ii*) 3-*x*, -*y*, 2-*z*; *Cg*1, *Cg*2, *Cg*3 and *Cg*4 are the centroids of the S1A/N1A/N2A/C4A/C5A, C1A–C6A, S1B/N1B/N2B/C4B/C5B and C1B–C6B rings, respectively]. These dimers are further tied together *via* pairs of N—H···N hydrogen-bonds with the dihydroimidazole rings as the hydrogen bonding donor (Table 1 and Fig. 3) and one of the benzothiadiazole N atoms as the acceptor, generating rings of graph set motifs of the type *R*^2^_2_(16) (Bernstein *et al.*, 1995). The A_2_ and B_2_ dimers do in turn form additional N—H···N hydrogen-bonds, with the secondary amine as the H donor and the dihydroimidazole N atom as the acceptor. These *R*^2^_2_(8) type interactions connect the A_2_ and B_2_ dimers with each other to form infinite chains that stretch along the (1 -1 1) direction of the unit cell (Fig. 3).

### Experimental

To 0.3 g of tizanidine in 10 ml methanol were added several drops of sodium hydroxide solution (3%) to adjust the pH to 8. The resulting solution was left for slow evaporation. Orange crystals were obtained after three days.

### Refinement

In the last cycles of the refinement, 24 reflections were eliminated due to being poorly measured in the vicinity of the beam stop. The H atoms of the NH groups of the molecules A and B were located in a difference map and refined with a distance restraint of N—H = 0.86 (3) Å. Their isotropic displacement parameters were set to be 1.2*U*_eq_(N). The HN3A···N5B distance was restrained to be 2.00 (2) Å. The other H atoms were positioned geometrically with C—H = 0.93 and 0.97 Å, and allowed to ride on their parent atoms, with *U*_iso_(H) = 1.2*U*_eq_(C).

### Crystal data

**Table:**

C_9_H_8_ClN_5_S	*Z* = 4
*M**_r_* = 253.72	*F*(000) = 520
Triclinic, *P*1	*D*_x_ = 1.593 Mg m^−^^3^
Hall symbol: -P 1	Mo *K*α radiation, λ = 0.71073 Å
*a* = 7.6927 (3) Å	Cell parameters from 9959 reflections
*b* = 10.8558 (4) Å	θ = 2.9–28.3°
*c* = 12.9969 (5) Å	µ = 0.54 mm^−^^1^
α = 95.790 (1)°	*T* = 296 K
β = 101.126 (1)°	Block, orange
γ = 92.192 (1)°	0.29 × 0.18 × 0.08 mm
*V* = 1057.69 (7) Å^3^	

### Data collection

**Table:**

Bruker APEXII CCD diffractometer	4449 reflections with *I* > 2σ(*I*)
Radiation source: sealed tube	*R*_int_ = 0.024
graphite	θ_max_ = 28.3°, θ_min_ = 3.2°
φ and ω scans	*h* = −10→10
17897 measured reflections	*k* = −14→14
5104 independent reflections	*l* = −17→17

### Refinement

**Table:**

Refinement on *F*^2^	Primary atom site location: structure-invariant direct methods
Least-squares matrix: full	Secondary atom site location: difference Fourier map
*R*[*F*^2^ > 2σ(*F*^2^)] = 0.042	Hydrogen site location: inferred from neighbouring sites
*wR*(*F*^2^) = 0.120	H atoms treated by a mixture of independent and constrained refinement
*S* = 1.03	*w* = 1/[σ^2^(*F*_o_^2^) + (0.0635*P*)^2^ + 0.4931*P*] where *P* = (*F*_o_^2^ + 2*F*_c_^2^)/3
5104 reflections	(Δ/σ)_max_ = 0.001
302 parameters	Δρ_max_ = 0.65 e Å^−^^3^
5 restraints	Δρ_min_ = −0.43 e Å^−^^3^

### Special details

**Table:**

Geometry. Bond distances, angles *etc*. have been calculated using the rounded fractional coordinates. All su's are estimated from the variances of the (full) variance-covariance matrix. The cell e.s.d.'s are taken into account in the estimation of distances, angles and torsion angles
Refinement. Refinement on *F*^2^ for ALL reflections except those flagged by the user for potential systematic errors. Weighted *R*-factors *wR* and all goodnesses of fit *S* are based on *F*^2^, conventional *R*-factors *R* are based on *F*, with *F* set to zero for negative *F*^2^. The observed criterion of *F*^2^ > σ(*F*^2^) is used only for calculating -*R*-factor-obs *etc*. and is not relevant to the choice of reflections for refinement. *R*-factors based on *F*^2^ are statistically about twice as large as those based on *F*, and *R*-factors based on ALL data will be even larger.

### Fractional atomic coordinates and isotropic or equivalent isotropic displacement parameters (Å^2^)

**Table:**

	*x*	*y*	*z*	*U*_iso_*/*U*_eq_	
Cl1A	0.40681 (8)	0.26166 (5)	0.72358 (5)	0.0597 (2)	
S1A	0.85136 (8)	0.69598 (5)	0.58255 (5)	0.0609 (2)	
N1A	0.6430 (3)	0.71482 (15)	0.57232 (14)	0.0518 (5)	
N2A	0.8692 (2)	0.56543 (15)	0.63157 (15)	0.0504 (5)	
N3A	0.7911 (2)	0.33724 (14)	0.71346 (12)	0.0441 (5)	
N4A	0.6996 (2)	0.18129 (15)	0.56630 (12)	0.0426 (4)	
N5A	0.9113 (2)	0.14570 (13)	0.69909 (11)	0.0393 (4)	
C1A	0.4819 (3)	0.39656 (17)	0.68103 (13)	0.0410 (5)	
C2A	0.3519 (3)	0.48262 (19)	0.65002 (14)	0.0449 (6)	
C3A	0.3954 (3)	0.58968 (17)	0.61257 (15)	0.0452 (5)	
C4A	0.5753 (3)	0.61502 (15)	0.60785 (13)	0.0407 (5)	
C5A	0.7058 (2)	0.52907 (15)	0.64166 (13)	0.0378 (5)	
C6A	0.6584 (2)	0.41428 (15)	0.67789 (13)	0.0375 (5)	
C7A	0.7965 (2)	0.22922 (15)	0.66323 (12)	0.0333 (4)	
C8A	0.7270 (3)	0.0496 (2)	0.55046 (19)	0.0588 (7)	
C9A	0.9066 (3)	0.0408 (2)	0.62174 (16)	0.0526 (6)	
Cl1B	1.12566 (6)	0.18138 (5)	1.13956 (4)	0.0505 (1)	
S1B	1.35483 (8)	−0.13008 (5)	0.75206 (4)	0.0509 (2)	
N1B	1.3845 (2)	−0.19353 (14)	0.86079 (14)	0.0469 (5)	
N2B	1.2747 (2)	−0.00089 (13)	0.78769 (11)	0.0380 (4)	
N3B	1.13963 (18)	0.19625 (12)	0.90223 (11)	0.0321 (3)	
N4B	1.32552 (18)	0.36020 (13)	1.01366 (12)	0.0352 (4)	
N5B	1.09790 (19)	0.40512 (13)	0.89434 (12)	0.0388 (4)	
C1B	1.2069 (2)	0.07385 (17)	1.05470 (13)	0.0359 (5)	
C2B	1.2668 (2)	−0.03721 (19)	1.09644 (15)	0.0452 (6)	
C3B	1.3290 (2)	−0.12898 (18)	1.03814 (16)	0.0454 (5)	
C4B	1.3311 (2)	−0.11275 (15)	0.93194 (14)	0.0367 (4)	
C5B	1.26880 (19)	−0.00179 (14)	0.88987 (12)	0.0306 (4)	
C6B	1.20705 (18)	0.09766 (14)	0.95246 (12)	0.0288 (4)	
C7B	1.18549 (19)	0.31124 (14)	0.93720 (12)	0.0297 (4)	
C8B	1.3016 (2)	0.49159 (16)	1.03987 (15)	0.0406 (5)	
C9B	1.1930 (2)	0.52377 (16)	0.93606 (16)	0.0424 (5)	
H8AA	0.73030	0.02310	0.47730	0.0710*	
H8BA	0.63520	0.00040	0.57190	0.0710*	
H2A	0.23490	0.46520	0.65550	0.0540*	
H9AA	0.91290	−0.03620	0.65370	0.0630*	
H3A	0.30950	0.64460	0.59070	0.0540*	
H9BA	1.00250	0.04780	0.58350	0.0630*	
HN3A	0.887 (3)	0.368 (3)	0.7644 (17)	0.0720*	
HN4A	0.597 (3)	0.209 (3)	0.544 (2)	0.0720*	
H2B	1.26270	−0.04660	1.16630	0.0540*	
H3B	1.36890	−0.20030	1.06680	0.0550*	
H8AB	1.41440	0.53940	1.05880	0.0490*	
H8BB	1.23730	0.50460	1.09690	0.0490*	
H9AB	1.11220	0.58780	0.94760	0.0510*	
H9BB	1.26870	0.55020	0.88970	0.0510*	
HN3B	1.072 (3)	0.179 (2)	0.8391 (15)	0.0610*	
HN4B	1.370 (3)	0.317 (2)	1.0608 (18)	0.0610*	

### Atomic displacement parameters (Å^2^)

**Table:**

	*U*^11^	*U*^22^	*U*^33^	*U*^12^	*U*^13^	*U*^23^
Cl1A	0.0697 (3)	0.0577 (3)	0.0579 (3)	0.0072 (2)	0.0179 (3)	0.0245 (2)
S1A	0.0653 (3)	0.0384 (3)	0.0738 (4)	−0.0043 (2)	0.0007 (3)	0.0089 (2)
N1A	0.0690 (11)	0.0306 (7)	0.0500 (9)	0.0073 (7)	−0.0036 (8)	0.0047 (7)
N2A	0.0487 (9)	0.0364 (8)	0.0591 (10)	0.0036 (6)	−0.0049 (7)	0.0019 (7)
N3A	0.0512 (9)	0.0320 (7)	0.0401 (8)	0.0131 (6)	−0.0144 (6)	0.0016 (6)
N4A	0.0459 (8)	0.0443 (8)	0.0320 (7)	0.0146 (6)	−0.0074 (6)	0.0016 (6)
N5A	0.0450 (8)	0.0329 (7)	0.0348 (7)	0.0129 (6)	−0.0057 (6)	0.0016 (6)
C1A	0.0548 (10)	0.0375 (9)	0.0300 (8)	0.0107 (7)	0.0041 (7)	0.0054 (6)
C2A	0.0492 (10)	0.0483 (10)	0.0367 (9)	0.0146 (8)	0.0070 (7)	0.0002 (7)
C3A	0.0545 (10)	0.0386 (9)	0.0392 (9)	0.0227 (8)	−0.0003 (8)	−0.0008 (7)
C4A	0.0570 (10)	0.0274 (7)	0.0320 (8)	0.0130 (7)	−0.0048 (7)	−0.0017 (6)
C5A	0.0466 (9)	0.0286 (7)	0.0324 (8)	0.0089 (6)	−0.0053 (6)	−0.0021 (6)
C6A	0.0496 (9)	0.0305 (8)	0.0275 (7)	0.0119 (7)	−0.0054 (6)	0.0008 (6)
C7A	0.0368 (7)	0.0328 (7)	0.0286 (7)	0.0056 (6)	−0.0007 (6)	0.0082 (6)
C8A	0.0549 (12)	0.0512 (11)	0.0555 (12)	0.0199 (9)	−0.0183 (9)	−0.0148 (9)
C9A	0.0517 (11)	0.0503 (11)	0.0456 (10)	0.0224 (9)	−0.0109 (8)	−0.0116 (8)
Cl1B	0.0461 (2)	0.0679 (3)	0.0390 (2)	−0.0006 (2)	0.0148 (2)	0.0023 (2)
S1B	0.0633 (3)	0.0406 (3)	0.0434 (3)	0.0166 (2)	−0.0008 (2)	−0.0036 (2)
N1B	0.0479 (9)	0.0308 (7)	0.0561 (10)	0.0076 (6)	−0.0055 (7)	0.0050 (7)
N2B	0.0453 (8)	0.0328 (7)	0.0325 (7)	0.0074 (6)	−0.0015 (6)	0.0034 (5)
N3B	0.0340 (6)	0.0299 (6)	0.0288 (6)	0.0031 (5)	−0.0030 (5)	0.0034 (5)
N4B	0.0299 (6)	0.0325 (7)	0.0393 (7)	0.0007 (5)	−0.0014 (5)	0.0014 (5)
N5B	0.0375 (7)	0.0281 (6)	0.0467 (8)	0.0076 (5)	−0.0014 (6)	0.0007 (6)
C1B	0.0292 (7)	0.0442 (9)	0.0332 (8)	−0.0048 (6)	0.0033 (6)	0.0074 (7)
C2B	0.0396 (9)	0.0578 (11)	0.0386 (9)	−0.0080 (8)	0.0012 (7)	0.0236 (8)
C3B	0.0422 (9)	0.0414 (9)	0.0514 (10)	−0.0022 (7)	−0.0029 (8)	0.0246 (8)
C4B	0.0309 (7)	0.0293 (7)	0.0456 (9)	−0.0014 (6)	−0.0057 (6)	0.0100 (6)
C5B	0.0280 (7)	0.0273 (7)	0.0330 (7)	−0.0019 (5)	−0.0034 (5)	0.0065 (6)
C6B	0.0243 (6)	0.0293 (7)	0.0304 (7)	−0.0020 (5)	−0.0012 (5)	0.0056 (5)
C7B	0.0265 (6)	0.0311 (7)	0.0312 (7)	0.0037 (5)	0.0052 (5)	0.0016 (6)
C8B	0.0336 (8)	0.0370 (8)	0.0477 (10)	−0.0001 (6)	0.0066 (7)	−0.0087 (7)
C9B	0.0403 (9)	0.0292 (8)	0.0560 (11)	0.0053 (6)	0.0071 (7)	0.0007 (7)

### Geometric parameters (Å, °)

**Table:**

Cl1A—C1A	1.734 (2)	C1A—C2A	1.424 (3)
Cl1B—C1B	1.7412 (18)	C1A—C6A	1.373 (3)
S1A—N2A	1.6114 (18)	C2A—C3A	1.359 (3)
S1A—N1A	1.605 (2)	C3A—C4A	1.415 (3)
S1B—N2B	1.6145 (16)	C4A—C5A	1.434 (3)
S1B—N1B	1.6148 (18)	C5A—C6A	1.434 (2)
N1A—C4A	1.345 (3)	C8A—C9A	1.520 (3)
N2A—C5A	1.338 (2)	C2A—H2A	0.9300
N3A—C7A	1.290 (2)	C3A—H3A	0.9300
N3A—C6A	1.384 (2)	C8A—H8AA	0.9700
N4A—C7A	1.375 (2)	C8A—H8BA	0.9700
N4A—C8A	1.453 (3)	C9A—H9BA	0.9700
N5A—C7A	1.344 (2)	C9A—H9AA	0.9700
N5A—C9A	1.436 (3)	C1B—C6B	1.379 (2)
N3A—HN3A	0.92 (2)	C1B—C2B	1.428 (3)
N4A—HN4A	0.86 (3)	C2B—C3B	1.349 (3)
N1B—C4B	1.343 (2)	C3B—C4B	1.412 (3)
N2B—C5B	1.339 (2)	C4B—C5B	1.434 (2)
N3B—C7B	1.296 (2)	C5B—C6B	1.437 (2)
N3B—C6B	1.376 (2)	C8B—C9B	1.524 (3)
N4B—C8B	1.460 (2)	C2B—H2B	0.9300
N4B—C7B	1.366 (2)	C3B—H3B	0.9300
N5B—C9B	1.457 (2)	C8B—H8AB	0.9700
N5B—C7B	1.351 (2)	C8B—H8BB	0.9700
N3B—HN3B	0.88 (2)	C9B—H9AB	0.9700
N4B—HN4B	0.84 (2)	C9B—H9BB	0.9700
			
N1A—S1A—N2A	101.46 (9)	N4A—C8A—H8BA	111.00
N1B—S1B—N2B	101.08 (8)	C9A—C8A—H8AA	111.00
S1A—N1A—C4A	105.99 (15)	C9A—C8A—H8BA	111.00
S1A—N2A—C5A	106.15 (13)	N4A—C8A—H8AA	111.00
C6A—N3A—C7A	120.01 (15)	H8AA—C8A—H8BA	109.00
C7A—N4A—C8A	108.65 (16)	H9AA—C9A—H9BA	109.00
C7A—N5A—C9A	111.44 (15)	C8A—C9A—H9BA	111.00
C6A—N3A—HN3A	120.0 (19)	C8A—C9A—H9AA	111.00
C7A—N3A—HN3A	118.9 (18)	N5A—C9A—H9AA	111.00
C8A—N4A—HN4A	121 (2)	N5A—C9A—H9BA	111.00
C7A—N4A—HN4A	119.2 (19)	Cl1B—C1B—C6B	119.74 (13)
S1B—N1B—C4B	106.09 (12)	C2B—C1B—C6B	123.76 (16)
S1B—N2B—C5B	106.29 (11)	Cl1B—C1B—C2B	116.49 (13)
C6B—N3B—C7B	123.72 (14)	C1B—C2B—C3B	122.33 (17)
C7B—N4B—C8B	108.92 (13)	C2B—C3B—C4B	117.52 (17)
C7B—N5B—C9B	110.47 (14)	C3B—C4B—C5B	120.03 (15)
C6B—N3B—HN3B	117.1 (14)	N1B—C4B—C3B	126.69 (16)
C7B—N3B—HN3B	118.8 (14)	N1B—C4B—C5B	113.27 (15)
C7B—N4B—HN4B	119.4 (15)	N2B—C5B—C6B	124.10 (14)
C8B—N4B—HN4B	120.4 (15)	C4B—C5B—C6B	122.63 (14)
C2A—C1A—C6A	124.01 (18)	N2B—C5B—C4B	113.27 (14)
Cl1A—C1A—C6A	119.56 (15)	N3B—C6B—C1B	128.30 (15)
Cl1A—C1A—C2A	116.43 (17)	N3B—C6B—C5B	117.62 (14)
C1A—C2A—C3A	121.3 (2)	C1B—C6B—C5B	113.69 (14)
C2A—C3A—C4A	117.99 (19)	N3B—C7B—N4B	129.45 (15)
N1A—C4A—C3A	126.55 (19)	N4B—C7B—N5B	108.75 (14)
N1A—C4A—C5A	113.2 (2)	N3B—C7B—N5B	121.71 (14)
C3A—C4A—C5A	120.22 (16)	N4B—C8B—C9B	101.13 (14)
C4A—C5A—C6A	121.60 (15)	N5B—C9B—C8B	101.10 (14)
N2A—C5A—C6A	125.17 (15)	C1B—C2B—H2B	119.00
N2A—C5A—C4A	113.17 (16)	C3B—C2B—H2B	119.00
N3A—C6A—C1A	125.98 (16)	C2B—C3B—H3B	121.00
N3A—C6A—C5A	118.99 (14)	C4B—C3B—H3B	121.00
C1A—C6A—C5A	114.83 (15)	N4B—C8B—H8AB	112.00
N3A—C7A—N5A	122.83 (15)	N4B—C8B—H8BB	112.00
N3A—C7A—N4A	128.44 (16)	C9B—C8B—H8AB	112.00
N4A—C7A—N5A	108.66 (14)	C9B—C8B—H8BB	112.00
N4A—C8A—C9A	102.08 (17)	H8AB—C8B—H8BB	109.00
N5A—C9A—C8A	101.67 (17)	N5B—C9B—H9AB	112.00
C1A—C2A—H2A	119.00	N5B—C9B—H9BB	112.00
C3A—C2A—H2A	119.00	C8B—C9B—H9AB	112.00
C2A—C3A—H3A	121.00	C8B—C9B—H9BB	112.00
C4A—C3A—H3A	121.00	H9AB—C9B—H9BB	109.00
			
N2A—S1A—N1A—C4A	−0.35 (15)	C2A—C1A—C6A—C5A	0.8 (2)
N1A—S1A—N2A—C5A	0.49 (16)	Cl1A—C1A—C2A—C3A	−178.11 (15)
N2B—S1B—N1B—C4B	−0.13 (13)	C6A—C1A—C2A—C3A	1.2 (3)
N1B—S1B—N2B—C5B	0.42 (13)	Cl1A—C1A—C6A—C5A	−179.89 (12)
S1A—N1A—C4A—C5A	0.10 (18)	C1A—C2A—C3A—C4A	−1.7 (3)
S1A—N1A—C4A—C3A	−179.03 (15)	C2A—C3A—C4A—C5A	0.2 (3)
S1A—N2A—C5A—C6A	176.96 (14)	C2A—C3A—C4A—N1A	179.23 (18)
S1A—N2A—C5A—C4A	−0.48 (19)	C3A—C4A—C5A—N2A	179.45 (17)
C6A—N3A—C7A—N4A	10.0 (3)	C3A—C4A—C5A—C6A	1.9 (3)
C7A—N3A—C6A—C5A	−115.36 (18)	N1A—C4A—C5A—N2A	0.3 (2)
C7A—N3A—C6A—C1A	70.1 (2)	N1A—C4A—C5A—C6A	−177.28 (16)
C6A—N3A—C7A—N5A	−173.51 (15)	C4A—C5A—C6A—N3A	−177.48 (15)
C8A—N4A—C7A—N3A	−169.83 (18)	N2A—C5A—C6A—C1A	−179.54 (17)
C7A—N4A—C8A—C9A	−24.8 (2)	C4A—C5A—C6A—C1A	−2.3 (2)
C8A—N4A—C7A—N5A	13.3 (2)	N2A—C5A—C6A—N3A	5.3 (3)
C7A—N5A—C9A—C8A	−20.2 (2)	N4A—C8A—C9A—N5A	26.1 (2)
C9A—N5A—C7A—N3A	−171.84 (17)	Cl1B—C1B—C6B—N3B	−4.3 (2)
C9A—N5A—C7A—N4A	5.3 (2)	C2B—C1B—C6B—C5B	1.7 (2)
S1B—N1B—C4B—C5B	−0.18 (17)	C6B—C1B—C2B—C3B	−0.1 (3)
S1B—N1B—C4B—C3B	178.69 (15)	Cl1B—C1B—C6B—C5B	−176.84 (11)
S1B—N2B—C5B—C6B	179.30 (13)	C2B—C1B—C6B—N3B	174.28 (16)
S1B—N2B—C5B—C4B	−0.57 (17)	Cl1B—C1B—C2B—C3B	178.50 (14)
C7B—N3B—C6B—C1B	55.9 (2)	C1B—C2B—C3B—C4B	−0.9 (3)
C7B—N3B—C6B—C5B	−131.72 (16)	C2B—C3B—C4B—N1B	−178.73 (17)
C6B—N3B—C7B—N5B	−170.11 (15)	C2B—C3B—C4B—C5B	0.1 (2)
C6B—N3B—C7B—N4B	13.9 (3)	N1B—C4B—C5B—N2B	0.5 (2)
C8B—N4B—C7B—N5B	14.92 (18)	N1B—C4B—C5B—C6B	−179.36 (14)
C7B—N4B—C8B—C9B	−28.11 (16)	C3B—C4B—C5B—N2B	−178.44 (15)
C8B—N4B—C7B—N3B	−168.70 (16)	C3B—C4B—C5B—C6B	1.7 (2)
C7B—N5B—C9B—C8B	−22.90 (17)	C4B—C5B—C6B—C1B	−2.5 (2)
C9B—N5B—C7B—N3B	−170.68 (15)	N2B—C5B—C6B—C1B	177.67 (15)
C9B—N5B—C7B—N4B	6.04 (19)	C4B—C5B—C6B—N3B	−175.91 (14)
Cl1A—C1A—C6A—N3A	−5.1 (2)	N2B—C5B—C6B—N3B	4.2 (2)
C2A—C1A—C6A—N3A	175.59 (17)	N4B—C8B—C9B—N5B	29.36 (15)

### Hydrogen-bond geometry (Å, °)

**Table:**

*D*—H···*A*	*D*—H	H···*A*	*D*···*A*	*D*—H···*A*
N3A—HN3A···N5B	0.92 (2)	2.10 (2)	3.003 (2)	168 (3)
N4A—HN4A···N1A^i^	0.86 (3)	2.38 (3)	3.205 (3)	160 (2)
N3B—HN3B···N5A	0.88 (2)	1.98 (2)	2.864 (2)	177 (2)
N4B—HN4B···Cl1B	0.84 (2)	2.75 (2)	3.1927 (15)	114 (2)
N4B—HN4B···N1B^ii^	0.84 (2)	2.48 (2)	3.227 (2)	150 (2)

Symmetry codes: (i) −*x*+1, −*y*+1, −*z*+1; (ii) −*x*+3, −*y*, −*z*+2.
